# High glutamate levels in the bone marrow of multiple myeloma patients promote osteoclast formation: a novel target for osteolytic bone disease

**DOI:** 10.1038/s41375-025-02715-2

**Published:** 2025-07-28

**Authors:** Denise Toscani, Oxana Lungu, Martina Chiu, Chiara Maccari, Vincenzo Raimondi, Giuseppe Taurino, Massimiliano G. Bianchi, Matteo Scita, Benedetta Dalla Palma, Nicolas Thomas Iannozzi, Rosanna Vescovini, Mattia Dessena, Camilla Sitzia, Paola Storti, Roberta Andreoli, Ovidio Bussolati, Nicola Giuliani

**Affiliations:** 1 Laboratory of Hematology, Department of Medicine and Surgery, University of Parma, Parma, Italy; 2 Hematology and BMT Unit, “Azienda Ospedaliero-Universitaria di Parma”, Parma, Italy; 3 Laboratory of General Pathology, Department of Medicine and Surgery, University of Parma, Parma, Italy; 4 Centre for Research in Toxicology (CERT), University of Parma, Parma, Italy; 5 Laboratory of Industrial Toxicology, Department of Medicine and Surgery, University of Parma, Parma, Italy

**Keywords:** Myeloma, Cancer metabolism

## Abstract

Multiple Myeloma (MM) is a glutamine (Gln)-addicted cancer. Consequently, the MM bone microenvironment (BM) is characterized by lower Gln and higher glutamate (Glu) levels than those in pre-malignant monoclonal gammopathies. Such MM-dependent metabolic perturbation impairs osteoblast differentiation in the bone microenvironment, but its effect on osteoclast (OCL) bone resorption is still unknown. We first show that bone marrow mononuclear cells from MM patients release higher levels of Glu compared to those from patients with monoclonal gammopathy of undetermined significance (MGUS) or smoldering multiple myeloma (SMM). This increased Glu production correlates with elevated bone resorption activity. We then demonstrate that Glu stimulates OCL differentiation *via* the activation of NF-κB-NFATc1 pathway in low-Glu BM samples from pre-malignant patients but not in high-Glu samples of MM patients. Secondly, the early phase of OCL formation was associated with high Glu intracellular content and induction of the Glu transporter EAAT1. Consistently, the pharmacological inhibition of EAAT1 hinders OCL differentiation by blocking the RANKL-dependent signaling pathway and actin cytoskeleton reorganization. Overall, our data indicate that high Glu levels in MM bone marrow are involved in OCL formation, suggesting that targeting Glu transport may represent a novel approach for the prevention of osteolytic lesions in MM patients.

## Introduction

Metabolic reprogramming of cancer cells is an established hallmark that can be exploited in new therapeutic approaches. Such metabolic alterations, mainly resulting from the abnormal activation of oncogenes and loss of onco-suppressor genes, provide malignant cells with a fitness advantage [1]. Over the years, several studies have shed light on the metabolic changes that occur in multiple myeloma (MM) [2–4]. In addition to glucose as an energy source, myeloma cells consume a sizable amount of glutamine (Gln) via a process known as glutaminolysis, which converts Gln to glutamate (Glu) via glutaminase (GLS1). Our group has previously demonstrated that malignant plasma cells (PCs) are highly dependent on extracellular Gln because of the lack of Gln synthetase (GS) [5]. MM cells metabolize large amounts of Gln to fulfill their energy and biosynthetic requirements and show increased activity of the Gln transporter ASCT2. Due to these alterations, the bone marrow (BM) of MM patients is characterized by lower Gln and higher levels of its metabolite Glu compared to patients with monoclonal gammopathy of undetermined significance (MGUS) and smoldering multiple myeloma (SMM) [5]. MM-dependent Gln depletion from the extracellular space adversely affects the cell populations in the bone microenvironment and is linked to the bone loss typical of MM patients [6]. In MM, the impairment of osteoblasts (OBs) leads to unbalanced and uncoupled bone remodeling towards the osteoclastic direction [7–9]. As shown by our group, high Gln consumption by MM cells causes GS induction in mesenchymal stromal cells (MSCs) and inhibits OBs differentiation by affecting osteogenic matrix biosynthesis in OB precursors [6]. Conversely, the impact of these alterations, particularly decreased Gln levels and increased Glu levels, on osteoclasts (OCLs) is still poorly understood. Glu is a non-essential amino acid that is the main excitatory neurotransmitter in the central nervous system, where Glu levels and signaling are precisely regulated by a complex network of receptors and transporters [10]. The ultimate step of regulation is mediated by Glu active uptake accomplished by Na^+^-dependent Excitatory Amino Acid Transporters (EAATs) [11]. Although described to be greater in the central nervous system, all of the machinery essential for Glu signaling and transport has been identified in non-neural tissues [12]. In bone, several previous studies have suggested that Glu signaling is involved in cell differentiation and mature cell activities [13–18]. Various Glu receptors and transporters are expressed by bone cells, in particular by OCLs and OBs, to ensure a tight modulation of Glu concentration within bone microenvironment [13]. Moreover, there is evidence that Glu signaling is involved in osteoclastic differentiation and bone resorption in non-human models [15–21]. However, the possible role of Glu in MM-induced OCL formation is still unknown to date.

Given this evidence, we explored whether changes in Gln metabolism, especially the elevated Glu levels observed in the MM bone microenvironment, contribute to the increased OCL activity characteristic of MM patients.

## Material and methods

### Patients

A total of 146 patients were included in the study: 27 with MGUS, 54 with SMM, and 65 with MM. All the patients included in the study provided written informed consent, consistent with the Declaration of Helsinki. All the study protocols were approved by the Ethics Committee “Area Vasta Emilia Nord” of Regional Health Service, Italy (protocol code 12/2017/SPER/AOUPR n. 7024). BM aspirates (treated with EDTA to prevent clotting) were obtained from the iliac crest of patients.

### Chemicals and reagents

Chemicals and reagents are described in the Supplementary Methods.

### Cells and cell culture conditions

#### Generation of conditioned medium from mononuclear cells (MNCs)

Conditioned medium (CM) was obtained as follow: total BM MNCs were obtained from BM aspirates of 5 patients with MGUS, 18 patients with SMM, and 21 patients with MM. Briefly, BM aspirate was layered over Lympholyte cell separation media (Cedarlane) and centrifugated at 800 × *g* for 30 min room temperature. The cell layer on top of the separating media was collected and washed twice with phosphate-buffered saline (PBS). Then, the cells were counted under light microscopy and cultured for 72 h at 37 °C in low-glucose DMEM supplemented with 5% FBS and 2 mM Gln, at a cell density of 500,000 cells/ml. At the end of the incubation, the conditioned media was collected by centrifuging the cell suspension at 300 × *g* for 10 min to remove cells and debris and stored at −20 °C. In a subset of samples, we purified the CD138+ cells from the MNCs fraction by immunomagnetic method with anti-CD138 mAb conjugated with microbeads (Miltenyi Biotech). The effective depletion has been verified for each sample by assessing the percentage of CD138+ before and after the depletion by flow cytometry. Both the CD138+ and CD138- fractions were cultured as described above. Glu concentration in the medium was checked by Glutamate Assay Kit (#ab83389 Abcam) following the manufacturer’s protocol.

#### Isolation of primary CD14^+^ cells from BM samples

MNCs were collected as described above. CD14^+^ monocytes were purified from BM MNCs by immunomagnetic method with anti-CD14 mAb conjugated with microbeads (Miltenyi Biotech) from 25 patients with MM, 15 with SMM, and 8 with MGUS. The presence of potential contaminating cells in each fraction was evaluated by flow cytometry analysis, using the fluorescence-activated flow cytometer BD Bioscience FACSCelesta flow cytometer using the FACSDiva Software (version 8.02, RRID:SCR_001456, BD Bioscience).

#### Osteoclast differentiation assay

OCLs were generated by incubating primary CD14^+^ monocytes in low-glucose DMEM containing 5% FBS and 2 mM of Gln, supplemented with recombinant human rhRANKL (60 ng/ml) and rhM-CSF (25 ng/ml) (Peprotech) in the presence or absence of Glu. Cells were differentiated for 12–15 days with the medium renewed every 2 days by aspirating the exhausted media and adding fresh media. At the end of the culture, OCLs were fixed with 4% paraformaldehyde/PBS (pH 7.4), and TRAP expression was examined by staining with a commercial kit (387A-1KT; Sigma-Aldrich Milan, Italy). OCLs were scored as multinucleated TRAP-positive cells (≥3 nuclei) and counted by microscopic observation with a AXIO Vert.A1 Zeiss microscope.

For Glu uptake and amino acid content determination, CD14^+^ cells were differentiated for 3 days and 8–14 days, respectively, in differentiating medium in the presence or absence of Glu. See below the detailed methods for Glu uptake and amino acid determination.

#### Glu inhibition during osteoclastogenesis

To analyze the effects of inhibitors of Glu transport on OCLs differentiation, primary CD14^+^ cells were seeded in 96-well plates in differentiating medium in the presence or absence of TFB-TBOA (1 µM), D-Asp (10 mM), or vehicle. The inhibitors were added at seeding for the entire culture period (12-15 days). At the end of culture, OCLs formation was assessed as described above from TRAP expression. Zoledronic acid (ZOL) at 10^−7^ M was used as a positive control of OCLs inhibition.

### Measurement of Glu transport

Anionic amino acid uptake was performed as described previously evaluating the 1-min influx of [^3^H]-Glu (10 µCi/ml, Perkin-Elmer) and counted with a scintillation spectrometer (Microbeta2, PerkinElmer, Milan, Italy) [22]. Data were expressed as pmol/mg protein/min after determination of cell proteins with a modified Lowry procedure.

### Measurement of amino acid content

Metabolite determination was performed as previously described [6] on OCLs differentiated from primary CD14^+^ cells. Briefly, cells were seeded in a 24-well plate. After 8 and 14 days of differentiation, cells were washed with ice-cold PBS, and metabolites were extracted with 0.15 mL absolute ethanol. LC analyses were carried out with UHPLC-MS/MS system (ExionLC-API 6500 +, Sciex).

### RNA isolation and real-time-polymerase chain reaction analysis

RNA Isolation and Real-Time-Polymerase Chain Reaction Analysis are described in the Supplementary Methods.

### Immunoblotting analysis

Immunoblotting analyses are described in the Supplementary Methods.

### Immunofluorescence staining

Primary CD14^+^ cells were grown on Ibidi 8-well μ-slide coated with ibiTreat (Ibidi, #80806) in differentiating medium in the presence or absence of Glu, TFB-TBOA, D-Asp and ZOL for 12–15 days with the media renewed every 2 days. Then, cells were fixed with 4% paraformaldehyde for 10 min, washed three times with PBS, and permeabilized in PBS containing 0.1% Triton X-100 for 15 min. Next, cells were incubated in blocking buffer (PBS containing 1% BSA) for 1 h at room temperature and subsequently exposed to primary antibody overnight at 4 °C. The following day, cells were incubated with a secondary antibody for 1 h and Alexa Fluor® 488 Phalloidin for 20 min. Cells were mounted in Fluoroshield™ with DAPI (#F6057, Sigma-Aldrich). The image capture was performed with AXIO Vert.A1 fluorescence microscopy (Zeiss) at different magnifications (2.5×, 10×, and 20×). The number of OCLs containing 3 or more nuclei and podosome belt structures were counted on at least 5 randomly selected fields/conditions.

Antibodies used were anti-EAAT1 (1:50, #AGC-021, RRID: AB_2039885, Alomone Labs) and Alexa Fluor® 546 anti-rabbit IgG (1:200, #A11010, RRID: AB_2534077, Life Technologies).

### BM CTX-1 and Glu levels

Soluble C-terminal telopeptide of collagen type I (CTX-I) and Glu in serum of primary BM samples (20 patients with MGUS, 32 patients with SMM and 43 patients with newly diagnosed MM (NDMM)) were detected by CrossLaps® (CTX-I) ELISA kit (Immunodiagnosticsystems, UK) and Glutamate Assay Kit (#ab138883, Abcam), respectively, following the manufacturers’ protocols.

### Statistics

Data were expressed as means ± standard deviation (SD). One-way ANOVA, Mann-Whitney, one-sample *t*-test, or paired/unpaired Student’s *t*-test were used to test significance. In all cases, *p* < 0.05 was considered significant. GraphPad Prism 8^TM^ (GraphPad Software Inc., RRID:SCR_002798, La Jolla, CA, USA) was used for all the statistical analyses. Correlation coefficients were quantified by the Spearman rank correlation coefficient. All experiments with representative images (western blot, TRAP, and immunofluorescence staining) were repeated at least twice, and representative images are shown.

## Results

### High Glu levels characterize BM MM and correlate with high bone turnover in patients

**Fig. 1:**
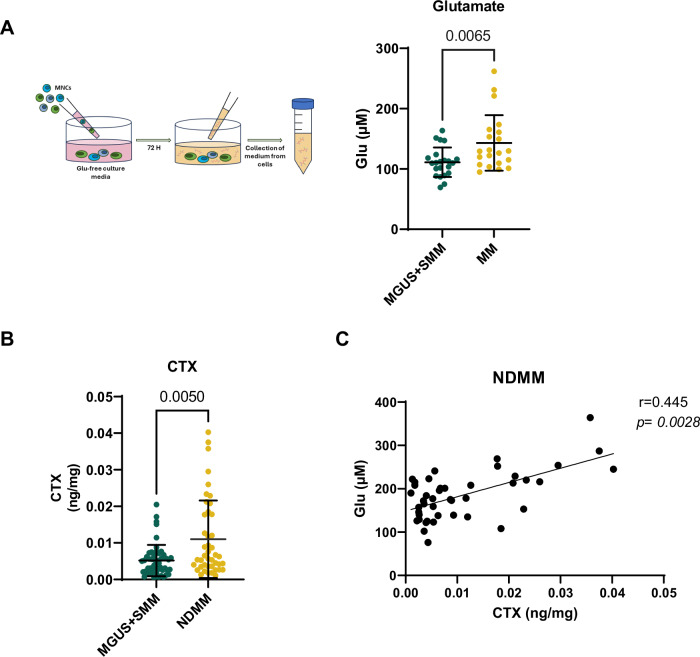
High Glu levels characterize BM MM and correlate with high bone turnover in patients. **A** Schematic illustration of the experimental procedure. Glu levels were assessed in CM collected from primary MNCs cultured for 72 h in Glu-free media (*n* = 5 patients with MGUS, *n* = 18 patients with SMM, and *n* = 21 MM). Data are presented as means ± SD. *P* = 0.0065 two-sided Student’s *t*-test for unpaired data. **B** CTX-1 levels measured in BM serum taken from patients with monoclonal gammopathies (*n* = 20 patients with MGUS, *n* = 32 patients with SMM, *n* = 43 patients with NDMM). Data are presented as means ± SD. ** *P* = 0.005, Mann-Whitney test. **C** Spearman correlation between the levels of CTX with those of Glu in the BM serum of MM patients (*r* = 0.445, *P* = 0.0028). Glu glutamate, BM bone marrow, MM multiple myeloma, CM conditioned medium, MNCs mononuclear cells, MGUS monoclonal gammopathy of undetermined significance, SMM smoldering MM, SD standard deviation, CTX C-terminal telopeptide of collagen type I, NDMM newly diagnosed MM.

Given our previous findings demonstrating high Glu levels in the BM of MM patients [5], we addressed the role of Glu in MM-induced OCLs formation. MNCs were obtained from patients with monoclonal gammopathies (5 patients with MGUS, 18 patients with SMM, and 21 patients with MM) and cultured in Glu-free culture media for 72 h following the procedures reported in Fig. 1A. CM were collected, and the Glu levels were measured as reported in Material and Methods. As shown in Fig. 1A, the CM of MM-MNCs have higher levels of Glu compared with that of MGUS- and SMM-MNCs, suggesting that BM MM was enriched of Glu, as previously reported by our group [5] (mean ± SD [Glu]: 111.2 ± 24.42 µM in MGUS + SMM; mean ± SD [Glu]: 143.2 ± 46.02 µM in MM, *P* = 0.0065, two-sided Student’s *t*-test for unpaired data). Interestingly, a positive correlation was found between the Glu levels and the % of BM PCs present at the time of the BM aspiration, as assessed by flow cytometry (Spearman *r* = 0.406, *P* = 0.0061, Supplementary Fig. 1A). To verify the contribution of PCs to Glu secretion, we measured the levels of Glu on purified CD138+ cells and CD138- fraction in a subgroup of patients. As shown in the Supplementary Fig. 1B, CD138+ cells produce higher levels of Glu compared to CD138- fraction, reflecting their high GLS1 expression (Supplementary Fig. 1C). Moreover, CM-CD138+ from MM patients have higher levels of Glu compared with those from SMM patients (Supplementary Fig. 1B).

To confirm the potential involvement of Glu in the development of osteolytic lesions, we measured the bone resorption marker CTX-I levels in the BM serum collected from a cohort of 95 patients (20 patients with MGUS, 32 patients with SMM, and 43 patients with NDMM). As expected, NDMM patients had higher levels of BM CTX-I than those with MGUS and SMM, reflecting the increased OCLs activity characteristic of MM patients (MGUS + SMM: 0.0051 ng/mg ± 0.004, NDMM: 0.011 ng/mg ± 0.010, Mann-Whitney test, *P* = 0.005) [23] (Fig. 1C). Interestingly, a positive correlation was found in NDMM samples between the levels of CTX-I and Glu levels measured in the same samples, suggesting that high Glu levels were associated with high bone turnover (Spearman *r* = 0.445, *P* = 0.0028) (Fig. 1D). Conversely, no significant correlation was found in samples of MGUS and SMM patients.

### Extracellular Glu stimulates OCLs formation in low-Glu BM samples of pre-malignant monoclonal gammopathies

**Fig. 2:**
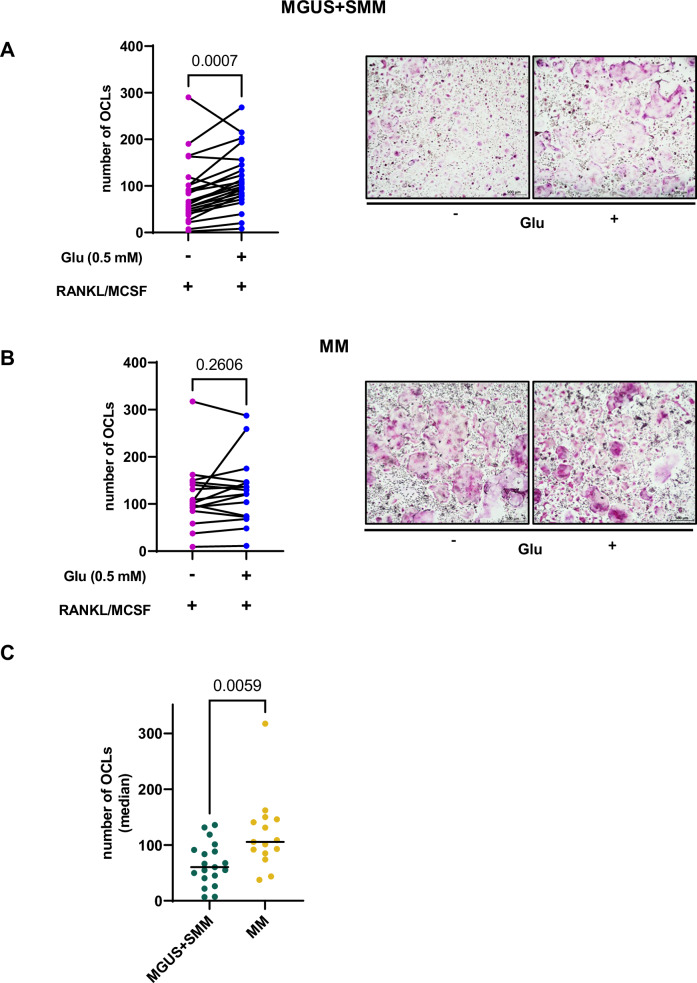
Extracellular Glu stimulates OCLs formation in pre-malignant samples. Primary CD14^+^ monocytes, isolated from MGUS and SMM (**A**) and MM patients (**B**), were cultured in differentiating medium in the presence or absence of Glu (0.5 mM) followed by TRAP staining. OCLs were scored as multinucleated TRAP-positive cells (≥3 nuclei) and counted by light microscopy. Graph represents the absolute number of OCL of each individual patient (MGUS + SMM *n* = 23, *P* = 0.0007 assessed by paired *t*-test; MM *n* = 16, *P* = 0.26 assessed by paired *t*-test) Right panel, Representative TRAP staining, for the identification of differentiated OCLs, performed at day 14 of incubation in the absence or in the presence of Glu, as indicated. Scale bar, 500 µm. **C** Number of OCLs obtained after 15 days of culture in standard medium in the absence of Glu. Graph represents the median number counted by light microscopy of each individual patient. *P* = 0.0059 as assessed two-sided Student’s *t*-test for unpaired data. Glu glutamate, OCLs osteoclasts, MGUS monoclonal gammopathy of undetermined significance, SMM smoldering multiple myeloma, MM multiple myeloma, TRAP Tartrate-Resistant Acid Phosphatase.

Based on these data, we aimed to understand the possible role of Glu in OCLs differentiation. For this purpose, CD14^+^ monocytes were purified from BM aspirates of patients with monoclonal gammopathies and cultured for 12–15 days in a medium containing the differentiating agents RANKL and M-CSF in the presence or absence of Glu. OCLs maturation was confirmed by TRAP staining. As shown in Fig. 2A, the presence of Glu significantly increased the number of OCLs generated from MGUS/SMM-derived CD14^+^ cells compared to cells differentiated in the absence of the amino acid (*P* = 0.0007, paired *t*-test). In contrast, CD14^+^ obtained from MM patients had a lower response to Glu, probably as a consequence of the in vivo exposure to the high-Glu microenvironment imposed by MM cells (Fig. 2B) [5]. Consistently, MM-derived CD14^+^ cells were prone to generate more OCLs in the absence of Glu compared with CD14^+^ cells obtained from premalignant samples (Fig. 2C) (median number of OCLs: MGUS + SMM 54, MM 108, *P* = 0.0059, two-sided Student’s *t*-test for unpaired data).

**Fig. 3:**
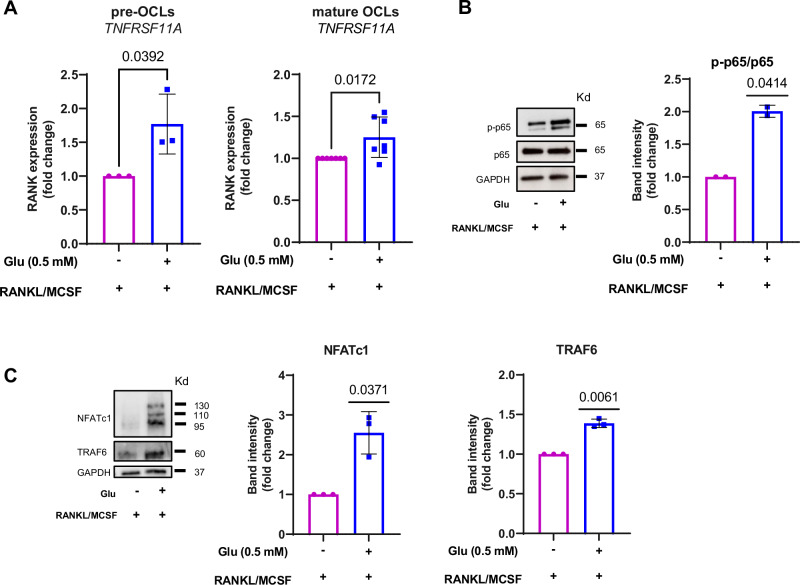
Glu stimulates OCL by regulating the RANK-TRAF6-NF-κB-NFATc1 pathway in pre-malignant gammopathies. **A** *TNFRSF11A* mRNA levels were assessed from pre-OCL and mature OCL differentiated in presence or absence of Glu (0.5 mM), as indicated. Graph represents the fold change relative to cells differentiated without Glu = 1 of each individual patient. Data are presented as means ± SD, (for pre-OCLs: *P* = 0.0392 *n* = 3, two-sided Student’s *t*-test for unpaired data; for mature OCLs: *P* = 0.0172 *n* = 6, two-sided Student’s *t*-test for unpaired data). **B**, **C** Western blot analysis of p-NF-κB, NF-κB, NFATc1, and TRAF6 in OCLs differentiated with or without Glu (0.5 mM). GAPDH was used as loading control. Band densitometry intensities represent the fold change relative to cells differentiated without Glu = 1 of each individual patient. Data are presented as means ± SD. (p-p65/p65: *P* = 0.0414 (*n* = 2), NFATc1: *P* = 0.0371 (*n* = 3), TRAF6: *P* = 0.0061 (*n* = 3), one sample *t*-test). Glu glutamate, OCL osteoclast, RANK Receptor Activator of Nuclear Factor κ B, TRAF TNF receptor associated factor, NF-κB nuclear factor kappa-light-chain-enhancer of activated B cells, NFATc1 Nuclear factor of activated T-cells, cytoplasmic 1, SD standard deviation, GAPDH glyceraldehyde-3-phosphate dehydrogenase.

We then examine the mechanistic basis of the effect of Glu on OCL formation. To mimic the high-Glu conditions of MM samples, we differentiated CD14^+^ cells, isolated from pre-malignant samples, MGUS and SMM, in the presence of Glu. Differentiation without the amino acid was used as a control. Activation of RANK by its ligand induces the expression of NFATc1, the main transcription regulator of OCL differentiation, through the recruitment of tumor necrosis factor receptor-associated factor 6 (TRAF6) and the activation of multiple signaling pathways such as NF-κB [24]. As shown in Fig. 3A, Glu increased RANK expression after either 3 days (pre-OCLs) or 14 days of differentiation (mature OCLs) (for pre-OCLs: *P* = 0.0392, two-sided Student’s *t*-test for unpaired data; for mature OCLs: *P* = 0.0172, two-sided Student’s *t*-test for unpaired data). In addition, Glu markedly increased the protein levels of TRAF6 as well as the phosphorylation of p65 (subunit of the NF-κB protein complex) and the level of NFATc1 (p-p65/p65: *P* = 0.0414, NFATc1: *P* = 0.0371, TRAF6: *P* = 0.0061, one sample *t*-test). (Fig. 3B, C). Collectively, our data suggest that Glu induces OCLs formation by stimulating the RANKL-induced activation of the RANK-TRAF6-NF-κB-NFATc1 pathway.

### Glu content and uptake increase during OCL differentiation

**Fig. 4:**
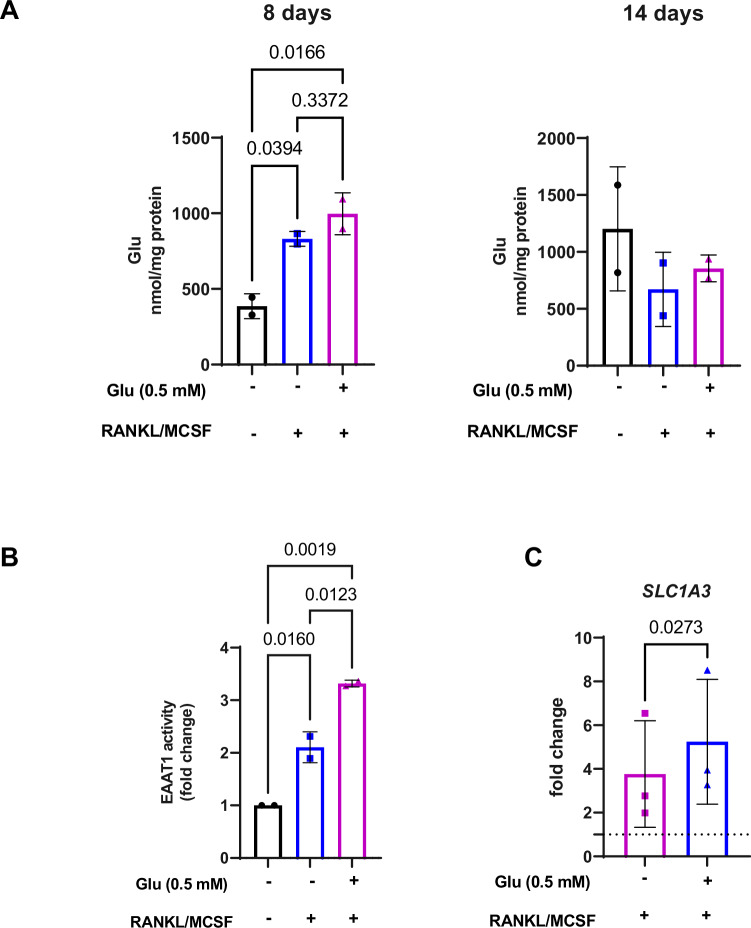
Glu Content and uptake increase during OCL differentiation. **A** Intracellular Glu content of primary CD14^+^ cells incubated for 8 and 14 days in standard (RANKL/MCSF, -) or differentiating medium (RANKL/MCSF, +) in the presence or absence of Glu (0.5 mM), as indicated. Data are means of two independent patients’ samples, three replicates per condition (differentiated without Glu vs. undifferentiated cells: *P* = 0.0394, differentiated with Glu vs. undifferentiated cells: *P* = 0.0166 one-way ANOVA). **B** Sodium-dependent ^3^H-Glu (10 µM, 10 µCi/ml) uptake was performed in primary CD14^+^ cells incubated for 3 days in standard (RANKL/MCSF, -) or differentiating (RANKL/MCSF, +) medium in the presence or absence of Glu (0.5 mM). Data are expressed as fold change relative to undifferentiated cells = 1 of two independent patients’ samples, five replicates per condition (differentiated without Glu *vs*. undifferentiated cells: *P* = 0.016, differentiated with Glu *vs*. undifferentiated cells: *P* = 0.0019, differentiated without Glu *vs*. differentiated cells with Glu: *P* = 0.0123 one-way ANOVA). **C** *SLC1A3* mRNA levels were assessed in CD14^+^ cells incubated for 3 days in standard (RANKL/MCSF, -) or differentiating (RANKL/MCSF, +) medium in the presence or absence of Glu (0.5 mM). Data are expressed as fold change relative to undifferentiated cells = 1 of three samples from different patients. (*P* = 0.0273, two-sided Student’s *t*-test for paired data). Glu glutamate, OCL osteoclast, RANKL Receptor Activator of Nuclear factor κB Ligand, MCSF macrophage colony-stimulating factor, SLC1A3 Solute Carrier Family 1 Member 3.

To assess the mechanism underlying the effect of Glu on OCLs formation, we measured the cell contents of Gln, Glu, and α-ketoglutarate (2-OG) in OCLs after 8 and 14 days of differentiation from MGUS/SMM-derived CD14^+^ cells. As shown in Fig. 4A, in pre-malignant OCLs, Glu content increased after 8 days of incubation in differentiating medium both in the presence or absence of extracellular Glu but decreased after 14 days indicating a fast utilization of Glu during the first phase of OCL formation (differentiated without Glu vs. undifferentiated cells: *P* = 0.0394, differentiated with Glu vs. undifferentiated cells: *P* = 0.0166, one-way ANOVA). The content of Gln and 2OG did not appreciably change throughout differentiation (data not shown).

These results suggest that OCL differentiation is stimulated by the availability of extracellular Glu and is associated with a rapid increase in the uptake of extracellular Glu upon osteoclastogenic stimuli. To verify the mechanism responsible for the accumulation of Glu, we analyzed the activity of the sodium-dependent Glu transporters EAATs, the major transporters for extracellular glutamate removal in the central nervous system [11]. As shown in Fig. 4B, EAATs activity had already increased after 3 days of incubation in a differentiating medium without Glu (differentiated without Glu vs. undifferentiated cells: *P* = 0.016, differentiated with Glu vs. undifferentiated cells: *P* = 0.0019, one-way ANOVA). Interestingly, when Glu was added to the culture media there was a significant increase in the transport activity compared to the condition without the amino acid (differentiated without Glu vs differentiated with Glu: *P* = 0.0123, one-way ANOVA).

We next performed OCL differentiation in the presence or absence of GLS1 inhibitor, CB-839 (1 µM) with or without Glu. As shown in the Supplementary Fig. 2A, we confirmed the significant increased levels of Glu during differentiation. Following GLS1 inhibition, the levels of Glu slightly decreased. Exposure to CB-839 in the presence of extracellular Glu, but not in the absence of the amino acid, increased the intracellular Glu levels compared to undifferentiated cells (differentiated without Glu vs. undifferentiated cells: *P* = 0.0002, differentiated with Glu vs. undifferentiated cells: *P* < 0.0001, differentiated with Glu vs differentiated with Glu + CB-839: *P* = 0.0029, differentiated with Glu + CB-839 vs undifferentiated cells: *P* = 0.0088, one-way ANOVA). This accumulation might be attributable to enhanced uptake of extracellular Glu by EAATs, rather than increased intracellular synthesis, consistently  with the inhibition of GLS1 activity by CB-839. Consistently, OCL differentiation was markedly inhibited by CB-839 without Glu. This effect was partly rescued by the addition of extracellular Glu as shown by TRAP staining (Supplementary Fig. 2B).

By real-time PCR screening of EAAT transporter family members (*SLC1A1-3, 6-7*), only *SCL1A3*, the gene for EAAT1, showed sizable expression levels. Consistent with the EAAT activity data, differentiation was associated with the induction of *SCL1A3*, with a more pronounced effect in the presence of Glu, suggesting EAAT1 involvement in Glu uptake by OCLs (*P* = 0.0273, two-sided Student’s *t*-test for paired data) (Fig. 4C). These observations are in line with a previous study demonstrating that EAAT1 is responsible for Glu transport by monocytes-derived macrophages, while other EAAT subtypes are barely detectable [25].

OCL differentiation is characterized by actin cytoskeleton reorganization, and mature OCLs contain characteristic actin structures such as clusters, rings, and a podosome belt [26]. Immunofluorescence microscopy confirmed that EAAT1 was expressed by mature OCLs and co-localizes with clusters, rings and podosome belts (Supplementary Fig. 3).

### Blocking Glu uptake through EAAT1 impairs OCL differentiation

We next investigated whether EAAT1 was critical for sustaining OCLs differentiation. EAAT1 inhibition was achieved *via* chemical and natural inhibitors, TFB-TBOA [27] and D-Asp [28], respectively, and the differentiation in the presence of Glu. ZOL at 10^−7 ^M was used as a positive control for OCL inhibition.

**Fig. 5:**
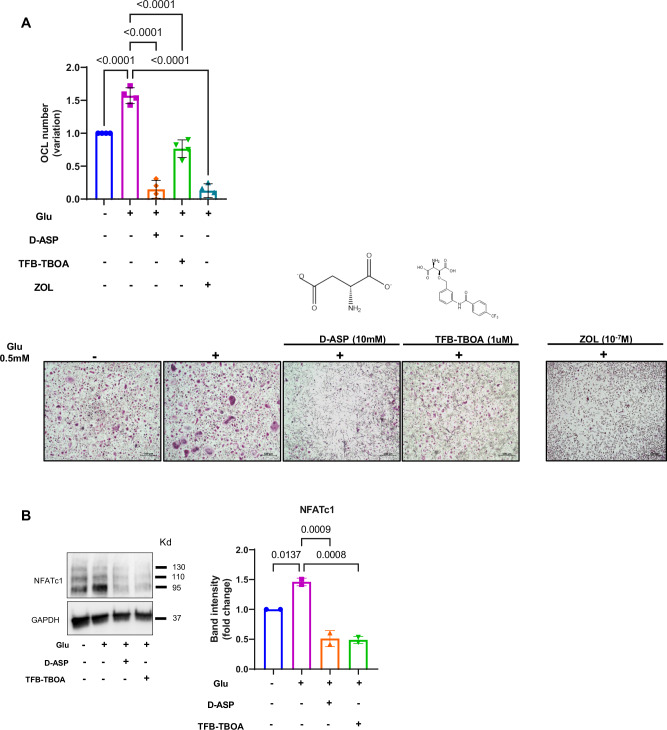
Blocking Glu uptake by EAAT1 impairs OCL differentiation. **A** left panel, CD14^+^ monocytes isolated from MGUS and SMM patients cultured in differentiating medium in the presence or absence of Glu (0.5 mM) and in the presence of Glu plus D-Asp (10 mM) or TFB-TBOA (1 µM) or vehicle, followed by TRAP staining. ZOL at 10^−7^ M was used as positive control for differentiation block. OCLs were scored as multinucleated TRAP-positive cells (≥3 nuclei) and counted by light microscopy. Graph represents the variation of OCL number relative to cells differentiated without Glu = 1 of each individual patient (*n* = 4). Data are presented as means ± SD. (−Glu *vs* +Glu: *P* < 0.0001; +Glu *vs* +Glu with D-Asp: *P* < 0.0001, +Glu *vs* +Glu with TFB-TBOA: *P* < 0.0001, +Glu *vs* +Glu with ZOL: *P* < 0.0001 one-way ANOVA). Right panel, Representative TRAP staining performed at day 14 of treatment for the identification of differentiated OCLs (−, Glu absent, + Glu present). Scale bars, 500 µm. **B** Western blot analysis of NFATc1 in OCLs differentiated in the absence or presence of Glu (0.5 mM), D-ASP (10 mM), or TFB-TBOA (1 µM). GAPDH was used as loading control. Band densitometry intensities represent the fold change relative to cells differentiated without Glu = 1 of two independent determinations. Data are presented as means ± SD. (−Glu vs +Glu: *P* = 0.0137; +Glu vs +Glu with D-Asp: *P* = 0.0009, +Glu vs +Glu with TFB-TBOA: *P* = 0.0008, one-way ANOVA). Glu glutamate, EAAT1 Excitatory amino acid transporter 1, OCL osteoclast, MGUS monoclonal gammopathy of undetermined significance, SMM smoldering multiple myeloma, D-Asp D-Aspartate, TFB-TBOA (3S)-3-[[3-[[4-(Trifluoromethyl)benzoyl]amino]phenyl]methoxy]-L-aspartic acid, TRAP Tartrate-Resistant Acid Phosphatase, ZOL: Zoledronic acid, SD standard deviation, NFATc1 Nuclear factor of activated T-cells, cytoplasmic 1, GAPDH glyceraldehyde-3-phosphate dehydrogenase.

**Fig. 6:**
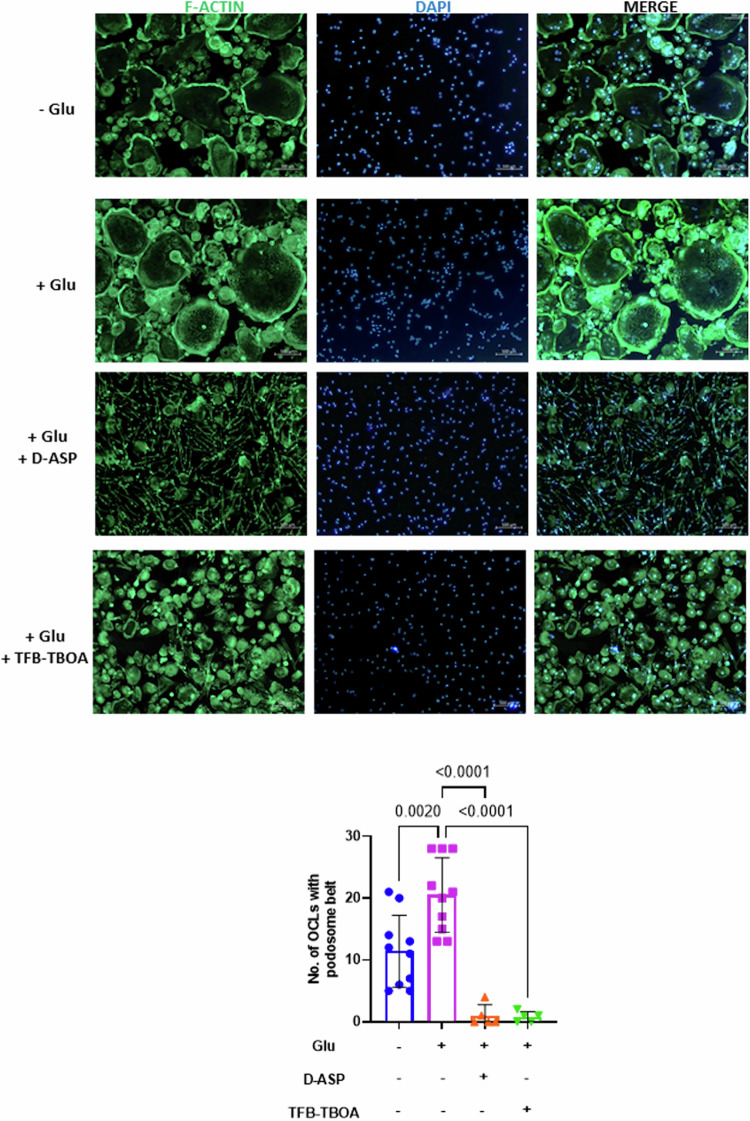
EAAT1 inhibition prevents actin cytoskeleton reorganization. CD14^+^ monocytes isolated from MGUS and SMM patients cultured in differentiating medium in the absence or presence of Glu (0.5 mM), D-Asp (10 mM), or TFB-TBOA (1 µM). After 14 days, cells were fixed and IF stained with Alexa Fluor® 488 Phalloidin (for F-actin) (green). Nuclei were counterstained with DAPI (blue). Scale bar, 500 μm. The number of OCLs containing 3 or more nuclei and with podosome belt structures were counted on at least 5 fields/condition randomly selected. The graph represents the mean ± SD. (−Glu vs +Glu: *P* = 0.002; +Glu vs +Glu with D-Asp: *P* < 0.0001, +Glu vs +Glu with TFB-TBOA: *P* < 0.0001, one-way ANOVA). EAAT1 Excitatory amino acid transporter 1, MGUS monoclonal gammopathy of undetermined significance, SMM smoldering multiple myeloma, Glu glutamate, D-Asp D-Aspartate, TFB-TBOA (3S)-3-[[3-[[4-(Trifluoromethyl)benzoyl]amino]phenyl]methoxy]-L-aspartic acid, IF immunofluorescence, DAPI diamidino-2-phenylindole, OCLs osteoclasts.

First, we confirmed the stimulatory effect of Glu on OCL formation and NFATc1 expression (Fig. 5A, B). Both inhibitors markedly inhibited the formation of multinucleated OCLs in the presence of Glu, which was comparable to that of ZOL (−Glu vs +Glu: *P* < 0.0001; +Glu vs +Glu with D-Asp: *P* < 0.0001, +Glu vs +Glu with TFB-TBOA: *P* < 0.0001, one-way ANOVA) (Fig. 5A). Consistent with these results, EAAT1 inhibition reduced NFATc1 levels even in the presence of differentiating medium (−Glu vs +Glu: *P* = 0.0137; +Glu vs +Glu with D-Asp: *P* = 0.0009, +Glu vs +Glu with TFB-TBOA: *P* = 0.0008, one-way ANOVA) (Fig. 5B). No significant effect on cell viability was observed after treatment with both inhibitors (data not shown). To examine the effect of inhibitors on actin cytoskeleton organization and cell nuclei, immunofluorescence was performed during differentiation in the presence or absence of inhibitors. As expected, the addition of Glu increased the number of OCLs forming the podosome belt, confirming the stimulatory effect on OCL formation (−Glu vs +Glu: *P* = 0.002; +Glu vs +Glu with D-Asp: *P* < 0.0001, +Glu vs +Glu with TFB-TBOA: *P* < 0.0001, one-way ANOVA). Interestingly, treatment with either D-Asp or TFB-TBOA completely prevented the formation of the podosome belt and the accumulation of nuclei (Fig. 6).

## Discussion

In this study, we showed that MM-induced metabolic alterations in the BM microenvironment influence OCL differentiation in patients with monoclonal gammopathies. Notably, the elevated Glu levels observed in MM patients correlate with increased bone turnover. Consistent with our previous study [5], we demonstrated that BM-MNCs from MM patients released more Glu that those from MGUS and SMM patients.

Moreover, we found that extracellular Glu sustains the formation of multinucleated OCL by stimulating the RANK-mediated pathway. We showed that osteoclastogenesis was characterized by increased Glu uptake by the transporter EAAT1, and that pharmacological inhibition completely blocked this differentiation. Overall, our findings highlight the critical role of metabolic alterations, particularly elevated levels of supraphysiological Glu, in promoting OCL formation and driving the progression of osteolytic bone disease. In the BM, interactions between MM cells and other BM cell populations are implicated in the pathogenesis of bone disease, which is sustained by an uncoupled and imbalanced bone remodeling. As a result, bone formation decreases while bone resorption increases, leading to the formation of osteolytic bone lesions [8]. We previously showed that malignant PCs depend on the massive uptake of extracellular Gln for their survival, thus lowering the levels of the amino acid in the BM plasma [5]. As a consequence of this Gln addiction, the level of Glu in BM plasma of MM was markedly increased compared with those found in patients with indolent monoclonal gammopathies MGUS and SMM [5]. This is in agreement with previous reports that an altered metabolic profile differentiated MGUS patients compared to MM [3, 29]. As demonstrated by our group, this low-Gln and high-Glu microenvironment, typical of MM, impaired OB differentiation of mesenchymal stromal cells, providing a metabolic mechanism underlying OB inhibition [6]. We demonstrate here that higher levels of Glu are present in the CM of cultured MM-derived MNCs compared with those from MGUS- and SMM-MNCs and positively correlate with the percentage of BM PCs, reflecting the active glutaminolysis characteristic of malignant PCs [5]. In a large cohort of NDMM patients, we have found that the serum levels of the bone resorption marker CTX-1 and Glu secretion were also positively correlated. As demonstrated by others, and confirmed in the current study, elevated CTX-1 levels distinguished NDMM patients [23]. Thus, we speculate that high Glu levels characterizes patients with high bone turnover and may be involved in increased OCL formation and bone resorption. Our hypothesis is supported by the increased levels of Glu present in arthritic patients [30], and by the association between circulating Glu and bone tissue resorption in patients with early rheumatoid arthritis [31].

In the present study, we specifically analyzed the effects of Glu during the differentiation of precursor cells isolated from high-Glu (MM) and low-Glu (MGUS-SMM) BM microenvironments. Here, we demonstrate that the addition of Glu during the differentiation of MGUS/SMM-derived CD14^+^ monocytes stimulate the formation of multinucleated cells. In contrast, the differentiation of MM-derived BM CD14^+^ cells were not affected by the addition of the amino acid. Moreover, MM-derived BM CD14^+^ were able to generate more OCL in vitro in the absence of Glu, consistent with previous works that demonstrated an increased number of OCL in MM samples compared with MGUS [32]. It is possible that MM-derived CD14^+^ are less sensitive to the presence of extracellular Glu in vitro due to the previous exposure to the high-Glu bone microenvironment of MM patients.

The involvement of glutamatergic signaling in normal bone homeostasis has been demonstrated in the past years with several models of osteoclastogenesis. Specifically, bone cells express receptors and transporters for Glu as well as the components of the involved signal transduction pathways [16, 19, 33]. Moreover, the involvement of Glu in bone homeostasis has been suggested in primary bone cancers [34] and in patients with rheumatoid arthritis [31], even though the mechanism remains unclear.

In this study, we used MGUS/SMM-derived CD14^+^ cells as a model to evaluate the mechanism underlying the Glu-dependent stimulation of OCL formation. We found that Glu administration during differentiation stimulated the RANK-TRAF6-NF-κB-NFATc1 pathway, the main signaling pathway required for the commitment of mononuclear precursors. These observations are supported by previous findings that Glu induced-NF-κB activation regulates OCL formation [33], a mechanism already demonstrated for neural cells [35]. Moreover, a more recent study have identified a specific metabolic signature of OCL differentiation consisting of increased abundance and uptake of Glu in mature OCL from mice [21]. A time-course study indicated a significant increase of intracellular Glu at the early phase of differentiation, while the decline observed once the maturation process was complete points to a fast uptake of the amino acid by early OCL precursors. Consistently, GLS1 inhibition in the presence of extracellular Glu, but not in the absence of the amino acid, still increased the intracellular Glu compared to undifferentiated cells, a process that might be attributable to enhanced uptake of extracellular Glu, rather than increased intracellular synthesis. We showed that the activity of the Glu transporters EAATs increased upon differentiation in the absence and, even at higher levels, in the presence of the amino acid. This increased activity was mediated by the induction of *SLC1A3*, which codes for the EAAT1 transporter. These observations are in line with a study performed on human blood monocytes demonstrating that EAAT1 expression increased at an early stage of culture coincident with the morphological differentiation of monocytes toward a macrophage-like phenotype [25]. The ability of Glu to promote its uptake *via* EAAT1 stimulation has already been demonstrated in astrocytes, where this effect was mediated by membrane translocation of the transporter involving actin cytoskeleton [36]. During OCL formation, the actin cytoskeleton undergoes an intensive rearrangement, ultimately leading to the formation of a podosome belt of actin, a critical structure for OCL migration, formation, and bone resorption [26, 37, 38].

In our model, Glu stimulated the formation of a podosome belt of actin, and EAAT1 co-localized with these membrane structures in mature OCLs, suggesting that these cytoskeleton modifications were functional to EAAT1 activity.

The EAAT1 promoter contains putative elements for both NF-kB and NFATc1 [39]. NF-κB is the main regulator of EAAT1 activity in astrocytes [40, 41]. In our system, we propose that the osteoclastogenic stimuli, such as Glu, which activates NF-κB and NFATc1, may stimulate EAAT1 in OCL precursors, providing a mechanism behind its transcriptional regulation during osteoclastogenesis. The role of EAAT1 during OCL differentiation is supported by the observation that its inhibition decreased NFATc1 levels resulting in an impairment of OCLs formation and a diminished number of OCLs forming podosome belts. We are the first to show that active Glu transport, specifically via the EAAT1 subtype, occurs in primary human OCL precursors and plays a role in regulating bone resorption in patients with monoclonal gammopathies.

From a translational perspective, elevated levels of Glu in patients with MM may reflect active remodeling of the bone microenvironment and could play a role in sustaining the osteolytic process characteristic of the disease. In addition to its metabolic functions, Glu may contribute to the dysregulated milieu that promotes OCL activation and enhances bone resorption.

Thus, targeting Glu or its associated metabolic pathways may offer a novel therapeutic approach to attenuate OCL-driven bone destruction and disrupt the metabolic cross-talk between MM cells and bone-resorbing OCLs. In this context, pharmacological inhibition of EAAT1 was sufficient to impair OCL formation by disrupting RANKL-mediated signaling, thereby targeting the tumor-altered metabolic pathways that drive osteoclastogenesis in the myeloma bone microenvironment.

These results suggest that targeting EAAT1 may represent a novel strategy that interferes with the metabolic support of osteoclastogenesis. Such an approach could potentially offer therapeutic synergy with standard anti-resorptive agents, especially in MM patients with high bone turnover driven by a Glu-rich microenvironment.

In summary, our study demonstrates that the increase in the levels of Glu in the BM of MM patients sustains OCL differentiation in vitro, supporting the hypothesis that the metabolic alterations induced by Gln-addicted MM cells underlie MM bone disease. We demonstrate that the BM of MM patients is enriched of Glu, whose levels correlate with bone turnover. Such high Glu levels stimulate OCL differentiation via activation of NF-κB pathway and increase the activity of the EAAT1 transporter, pointing to a role for Glu in supporting bone disease. The diminished OCL formation following EAAT1 inhibition supports previous studies, which suggested a possible involvement of Glu signaling in the skeletal system [14, 19, 33], and the possible exploitation of Glu transport as a novel therapeutic target in MM-induced bone resorption. Clearly, further work is needed to define the role of Glu in vivo to design novel therapeutic strategies to treat bone disease in MM.

## Supplementary information

Supplementary Information

## Data Availability

All data generated or analyzed during this study are included in this published article and its supplementary information files. For original data, please contact nicola.giuliani@unipr.it. The data generated in this study are available upon request from the corresponding authors.
