# Supplementary material for: High glutamate levels in the bone marrow of multiple myeloma patients promote osteoclast formation: a novel target for osteolytic bone disease
Source: Leukemia. 2025 Jul 28;39(10):2492–503. doi: 10.1038/s41375-025-02715-2 (PMC12463672; doi:10.1038/s41375-025-02715-2)
Supplement: Supplementary file 1 — Supplementary Information [file 41375_2025_2715_MOESM1_ESM.docx]

**Supplementary Materials and Methods for**

**High Glutamate Levels in The Bone Marrow of Multiple Myeloma Patients Promote Osteoclast Formation: A Novel Target for Osteolytic Bone Disease.**

Denise Toscani^1,2^, Oxana Lungu^1^, Martina Chiu^3^, Chiara Maccari^4^, Vincenzo Raimondi^1^, Giuseppe Taurino^3^, Massimiliano G. Bianchi^3^, Matteo Scita^2^, Benedetta Dalla Palma^2^, Nicolas Thomas Iannozzi^1^, Rosanna Vescovini^1^, Mattia Dessena^1^, Camilla Sitzia^1^, Paola Storti^1^, Roberta Andreoli^4,5^, Ovidio Bussolati^3^*, and Nicola Giuliani^1,2^*.

**Chemicals and reagents**

RPMI-1640, low-glucose Dulbecco modified minimal essential medium (DMEM) and other culture media, Gln, penicillin, streptomycin, and fetal bovine serum (FBS) were purchased from Invitrogen Life Technologies. Human recombinant RANKL (rhRANKL) and human recombinant M-CSF (rhM-CSF) were purchased from Peprotech, Rocky Hill, NJ.

CB-839 was purchased from Selleckchem (10mM in DMSO) (Catalog No.S7655).

The EAAT1 inhibitor (3S)-3-[[3-[[4-(trifluoromethyl)benzoyl]amino]phenyl]methoxy]-L-aspartic acid (TFB-TBOA) was purchased from Tocris Bioscience and resuspended in DMSO to 5mM.

Zoledronic acid (ZOL) was purchased from Sigma-Aldrich as hydrated disodium salt. Stock solution (50 mM) was prepared in phosphate-buffered saline (PBS), filter sterilized, and stored at –20°C until use.

**GLS1 inhibition during osteoclastogenesis**

To analyze the effects of GLS1 inhibitor on OCLs differentiation, primary CD14^+^ cells were seeded in 24- and 96-well plates in differentiating medium in the presence or absence of Glu and CB-839 (1µM), or vehicle. The inhibitor was added at seeding for the entire culture period (8 days). At the end of culture, OCLs formation was assessed as described above from TRAP expression. Intracellular Glu determination was assessed as described in the section “Measurement of Amino Acid Content”.

**RNA Isolation and Real-Time-Polymerase Chain Reaction Analysis**

Cells were previously suspended in RLT/β-mercaptoethanol buffer and, successively, total cellular RNA was extracted from cells using RNeasy total RNA isolation kit (Qiagen, Valencia, CA). Subsequently, extracted RNA was precipitated and resuspended with sterile water to obtain an optimal concentration (1μg/μL) and a better purity. Extracted RNA was quantified by Nanodrop ND-100 (Celbio S.p.A. Milan), and its purity was evaluated from A260/A280 ratio. Then, 0.5 μg of RNA was reverse-transcribed with 400 U Moloney murine leukemia virus reverse transcriptase (M-MuLV RT) according to the manufacturer’s protocol. Gene expression has been performed using TaqMan probe (Life Technologies) for the following genes:

*TNFRSF1*: Hs00921372_m1

*SCL1A2*: Hs01102423_m1

*SLC1A6*: Hs00192604_m1

*SCL1A7*: Hs00198515_m1

*SLC1A3*: Hs00188193_m1

*SLC1A1*: Hs00188172_m1

The Ct method was applied to normalize differences in the quantity and quality of RNA, and mRNA was quantified using the comparative ΔCt method and *GAPDH* as the endogenous reference gene. ΔCt was evaluated as the difference between the ΔCt value of the target sample and the ΔCt of the corresponding control sample. The fold change in mRNA expression was calculated as 2-ΔΔCt.

**Immunoblotting analysis**

Cells were collected and lysed using RIPA buffer (#156034, Abcam) including a protease inhibitor cocktail (Active Motif) and a phosphatase inhibitor cocktail (Active Motif) at 4°C for 30 minutes. Protein quantification was performed using the Bradford method (Bio-Rad protein assay) in triplicate and using a standard curve, a control sample (sterile water), and our samples diluted with sterile water.

Lysates were boiled to denature proteins, loaded onto SDS-PAGE gels for electrophoresis, and subsequently transferred to PVDF membranes (#1620177, BioRad Laboratories). Next, the membranes were blocked for 1 hour under agitation with a 5% milk- or BSA-blocking solution in TBS+0.1% Tween20 prior to overnight incubation with primary antibodies. The primary antibodies used were: Phospho-NF-κB p65 (1:1000 #3033, RRID: AB_331284), NF-κB p65 (1:1000 #8242, RRID: AB_10859369), GLS1 (1:1000 #49363) GAPDH (1:1000 #2118, RRID: AB_561053), all from Cell Signaling Technology; NFATc1 Antibody, clone 7A6 (#MABS409, RRID: AB_2152503) from Millipore; TRAF6 Antibody (1:500 # A16991, RRID:AB_2772697) from ABclonal. The next day, the membrane was washed 5 times in TBS-Tween, 5 minutes for wash. After washing, the membrane was incubated under agitation with a secondary antibody (Anti-rabbit IgG, HRP-linked Antibody #7074, RRID: AB_2099233 from Cell Signaling Technology or HRP Goat Anti-mouse IgG #554002 from BD Biosciences).

The secondary antibody was removed, and the membrane was washed 3 times with TBS-Tween, 5 minutes for wash. The chemiluminescence signals were developed using an ECL substrate at a ratio of 1:1 (Clarity Max Western ECL substrate #1705062, BioRad Laboratories) on a Chemidoc digital image acquisition machine (BioRad). Image quantification was performed using ImageJ software (RRID:SCR_003070, NIH).

**Supplementary Figure 1**


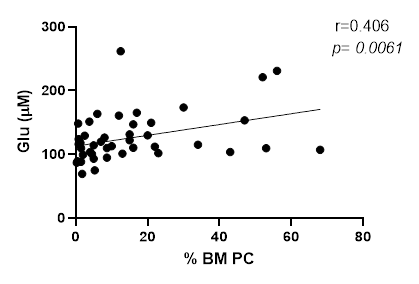
**A**

**B**

**
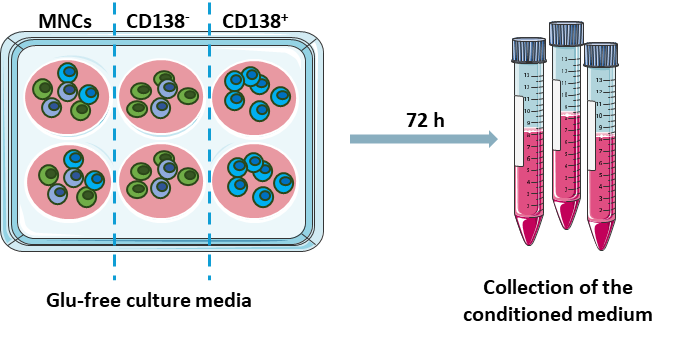
**


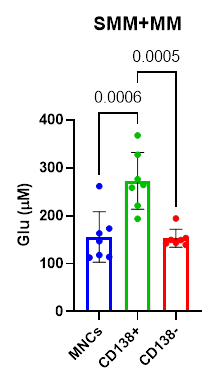


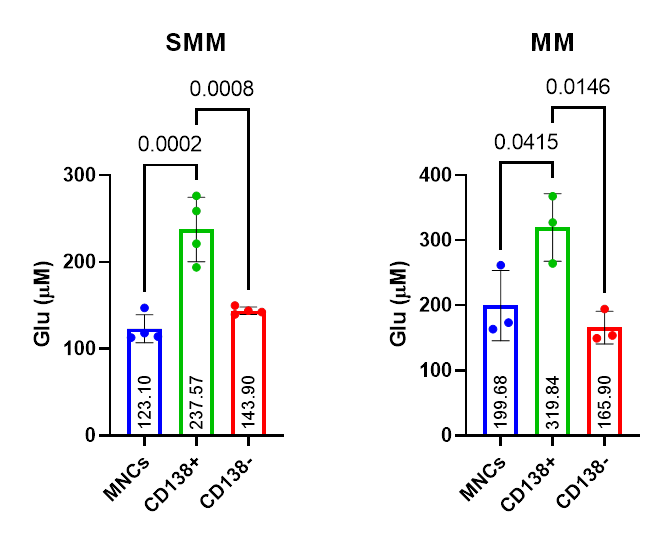


**C**


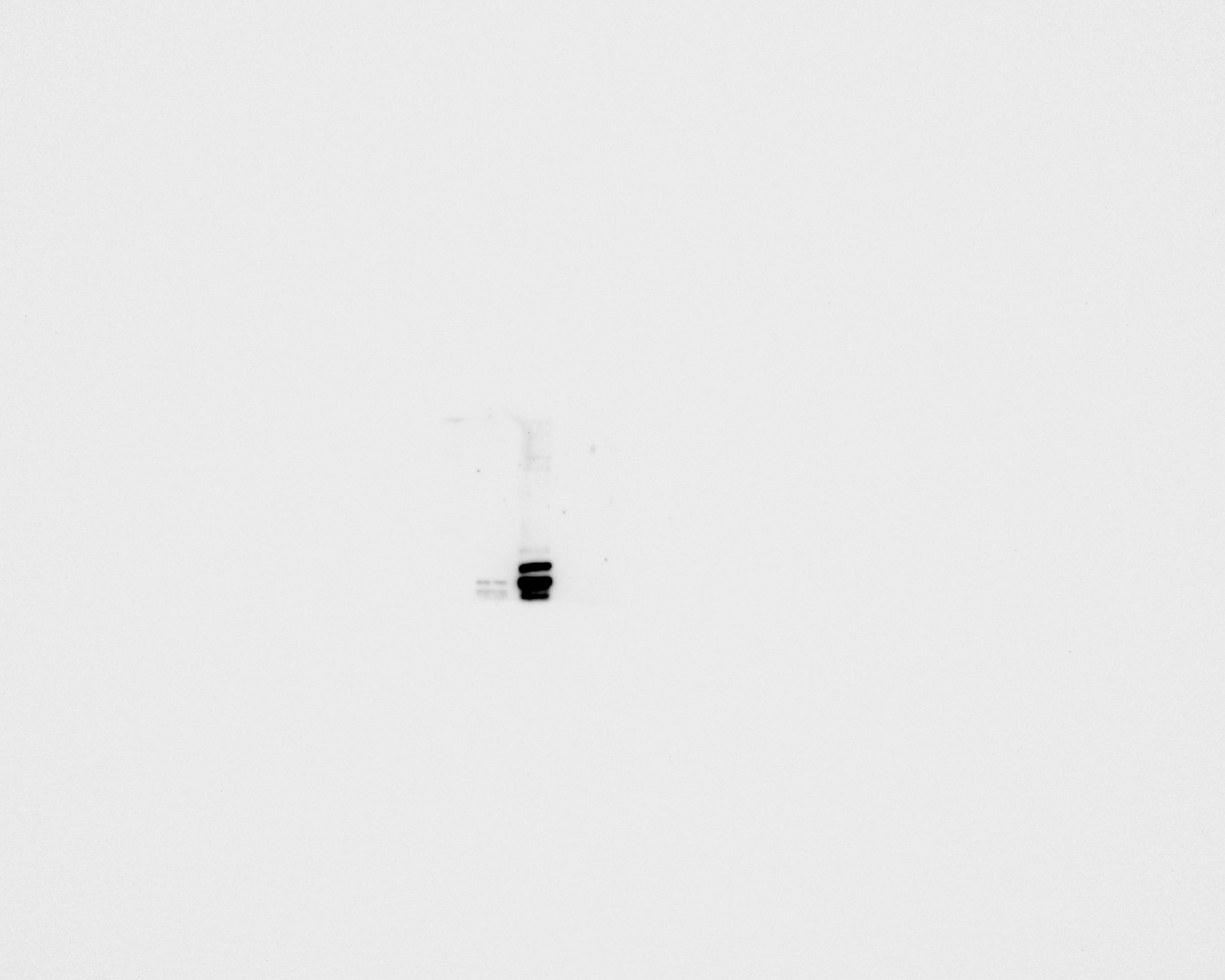

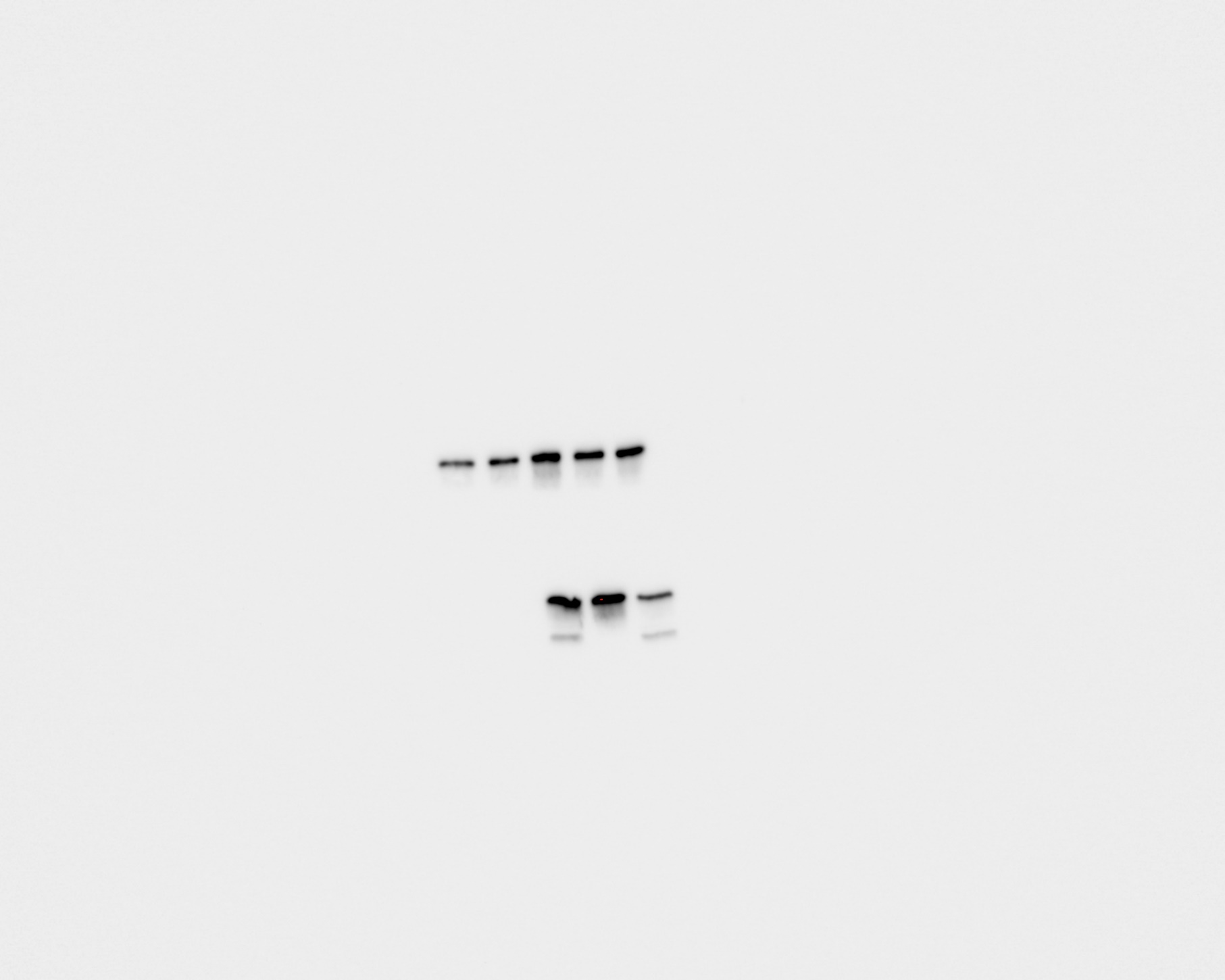


**MNCs**

**CD138^+^**

**CD138^-^**

**GLS1**

**GAPDH**

**Supplementary Figure 1: Glu levels in conditioned medium (CM) from primary samples**

**A.**Glu levels were assessed in CM collected from primary MNCs cultured for 72h in Glu-free media (n=5 patients with MGUS, n=18 patients with SMM, and n=21 MM). Spearman correlation between the levels of Glu in CM and the % of BM PC assessed in all samples (r= 0.406, P=0.0061). **B.** Schematic illustration of the experimental procedure. Glu levels were assessed in CM collected from primary MNCs, CD138- and CD138+ cell fraction cultured for 72h in Glu-free media (n=4 patients with SMM, and n=3 MM). Values within the bars represent the mean level of Glu. **C.** Western blot analysis of GLS1 in MNCS, CD138+ and CD138- cell fraction. GAPDH was used as loading control. The figure showed one representative patient of three.

Glu: glutamate, MNCs: mononuclear cells, MGUS: monoclonal gammopathy of undetermined significance, SMM: smoldering multiple myeloma, MM: multiple myeloma, BM: bone marrow, PC: plasma cell, GLS1: glutaminase 1, GAPDH: glyceraldehyde-3-phosphate dehydrogenase.

**Supplementary Figure 2**


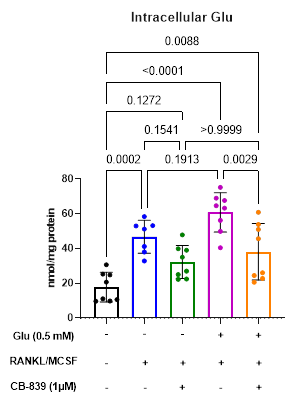
**A**


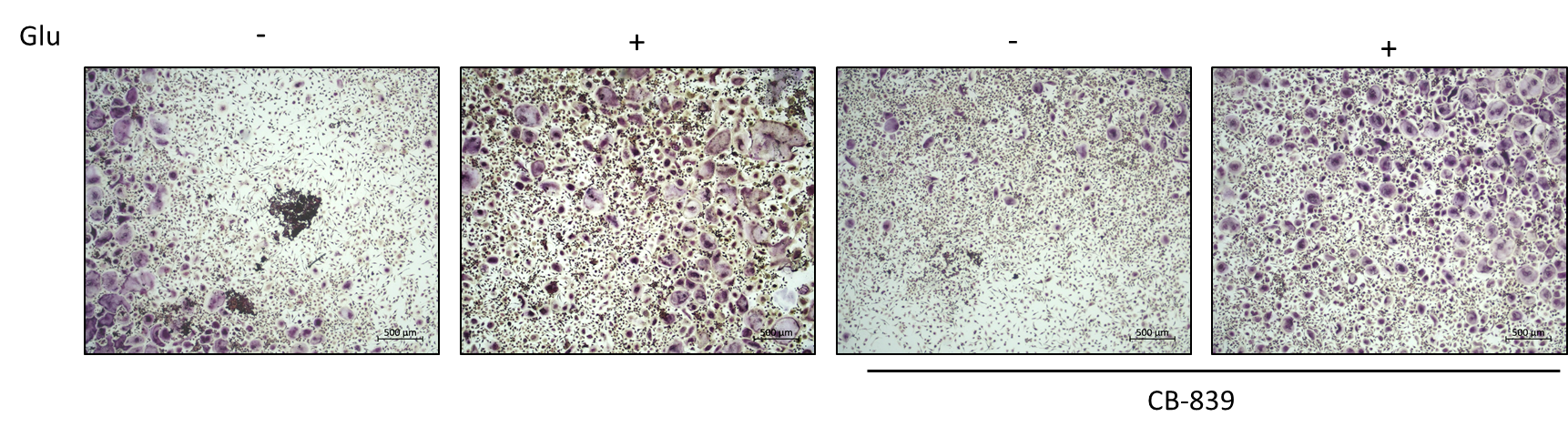
**B**

**Supplementary Figure 2: Extracellular Glu rescues the effects of GLS1 inhibition by** **CB-839**

**A.** Intracellular Glu content of primary CD14+ cells incubated for 8 days in standard (RANKL/MCSF, -) or differentiating medium (RANKL/MCSF, +) in the presence or absence of Glu (0.5mM) and CB-839 (1µM), as indicated. Data are means of two independent patients’ samples, four replicates per condition (differentiated without Glu vs. undifferentiated cells: P=0.0002, differentiated with Glu vs. undifferentiated cells: P<0.0001, differentiated with Glu vs differentiated with Glu + CB-839: P= 0.0029, differentiated with Glu + CB-839 vs undifferentiated cells: P= 0.0088, one-way ANOVA). **B.** Representative TRAP staining, for the identification of differentiated OCLs, performed at day 8 of incubation in the absence or in the presence of Glu and CB-839 (1µM), as indicated. Scale bar, 500 µm.

Glu: glutamate, GLS1: glutaminase 1, RANKL: Receptor Activator of Nuclear factor κB Ligand, MCSF: macrophage colony-stimulating factor, OCLs: osteoclasts.

**Supplementary Figure 3**

**
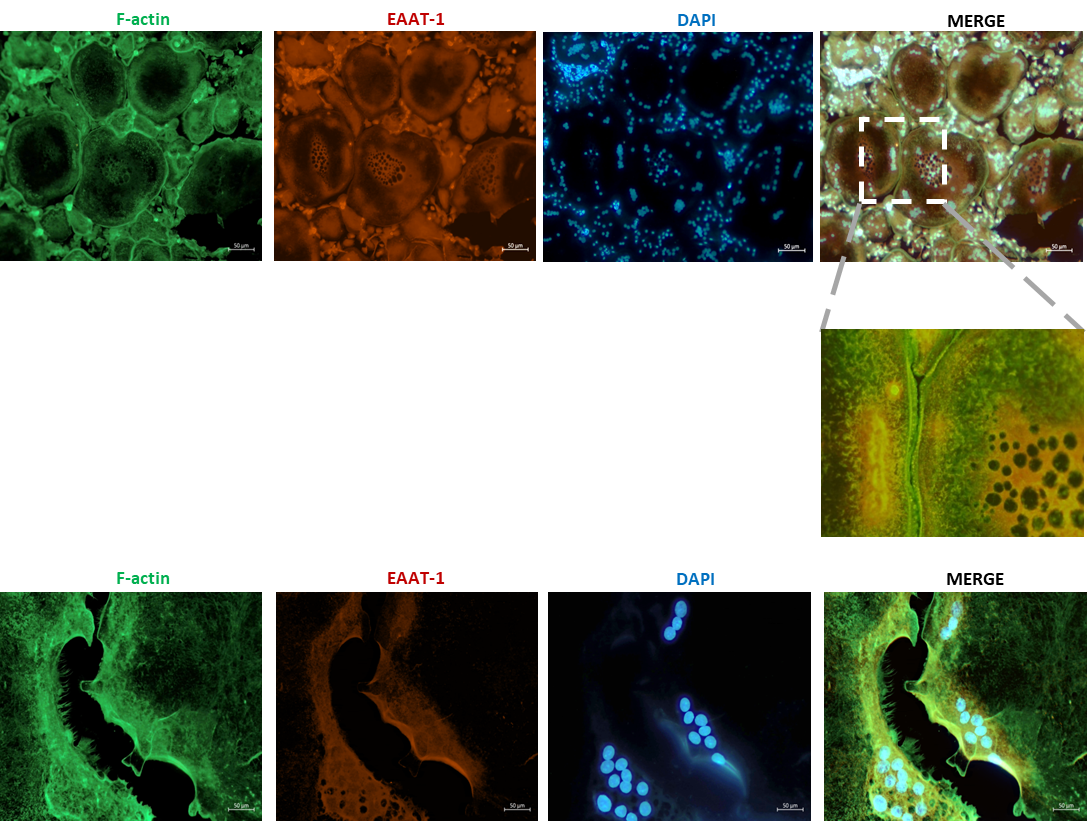
**

**Supplementary Figure 3: EAAT1 co-localizes with podosome belts in OCL.**

CD14^+^ monocytes isolated from MGUS and SMM patients were cultured in differentiating medium in the presence of Glu (0.5mM). After 14 days, cells were fixed and IF stained with Alexa Fluor® 488 Phalloidin (for F-actin) (green) and anti-EAAT1 followed by secondary antibody (Alexa Fluor® 546 anti-rabbit, orange). Nuclei were counterstained with DAPI (blue). Scale bar, 50 μm.

EAAT1: Excitatory amino acid transporter 1, OCL: osteoclast, MGUS: monoclonal gammopathy of undetermined significance, SMM: smoldering multiple myeloma, Glu: glutamate, DAPI: diamidino-2-phenylindole.
